# Treatment of Chronic Abscess of the Breast and Milk Fistulæ

**Published:** 1853-05

**Authors:** 


### OBSTETRICS.

*Treatment of Chronic Abscess of the Breast and Milk Fistulæ.—Employment of Iodine Injections.* [Cases under the care of Mr. BIRKETT.] —We have been much gratified by watching the result of the treatment pursued by Mr. Birkett in several cases of chronic inflammation of the breast, attended with sinuses and long continued suppuration, which have recently been under his care. This form of disease, as the result of neglected milk abscess, falls, we have no doubt, pretty frequently under the observation of most of our readers, and is often very troublesome to cure. The breast becomes affected with solid œdema, the sinuses, usually running in several directions through and behind the gland, are most difficult to close, and, by the constant discharge from them, the patient's powers become much undermined. In an extreme case of this description, which had lasted for many years, we not long ago saw Mr. South perform excision of the whole diseased mass, believing that to be the shortest method of getting rid of a useless part, which had become a serious evil to the patient. The remedies usually employed, are, as it is well known, support to the part, and the laying open freely of the sinuses, or the injection of them with various irritating fluids. Mr. Birkett's treatment consists in the employment of iodine taken internally, applied as an ointment over the tumor, and used also as an injection for the sinuses, whilst at the same time the part is carefully supported and subjected to gentle pressure by means of a bandage. The power of iodine, as a means of exciting the absorption of inflammatory products is well known; and, as an application to the lining membrane of sinous abscesses, it has for some time been employed on the Continent, and, less generally, in this country also. The success which has attended Mr. Birkett's method of treatment has been, as the following case will show, most encouraging.

Elizabeth Wiles, aged 26, the wife of a farm laborer, was admitted January 6th, 1853. Three years ago, while suckling, she suffered what from her description would appear to have been an attack of acute inflammation, both of the left mammary gland and of the surrounding structures. Her child was then eleven months old, and the disease appeared to have been excited by inflammation of the lymphatics of the arm from a sore on the finger. It was so severe as to confine her to bed for ten weeks, and to necessitate the employment of great number of leeches. It ultimately subsided considerably without having occasioned any abscess. The swelling, however, never quite disappeared, but after a time it again began to increase; and a year subsequent to the first attack, a large chronic abscess had to be opened, which never afterwards healed. In September, 1852, she was again confined. The disease of the breast having resisted the persevering treatment of several practitioners in the town where she lived, and her constitutional powers being evidently very much reduced by the long-continued and profuse discharge of pus which it produced, she was ultimately recommended to come up to town, in order to have Mr. Birkett's opinion as to the propriety of excision. On admission, she was emaciated, and of a somewhat hectic appearance; the discharge from the sinuses was very profuse, and the whole breast much indurated. She was then suckling with the right breast.

Mr. Birkett advised her to wean her infant, and ordered her to be confined to bed, with a poultice over the part for a few days, until the state of excessive irritation which appeared to exist had somewhat subsided. He then prescribed for her the following mixture :

℞. Potassii iodidi gr. iij., infus. gent. ℥i., ter die sumend.

The breast to be wrapped in lint spread with ung. plumb. iod., and the whole supported by a bandage carried round the shoulder. On the 23d, the symptoms having already begun to amend, the injection of all the sinuses with the tincture of iodine (London Pharmacopœia) was performed by means of a tube carried to the end of the sinus. The treatment as above was persevered in, and after the expiration of a week the injection was repeated, and again after another space of two weeks. At present, March 15, the sinuses are quite healed; the gland has been reduced very nearly to its natural size, and the patient has gained in general health to a point beyond what she has enjoyed since the commencement of the disease. With the exception of the first few days, she has been allowed to be out of bed the whole time, and full diet has been allowed her. On each occasion that the injection was used, it produced considerable smarting pain, and was followed by a temporary increase of swelling and discharge, which very quickly subsided.

We scarcely need point out the advantages of the above plan of treatment over the old method of laying open the sinuses. In the latter the incisions have to extend wide and deep, and they involve considerable hæmorrhage, and, for a time, increased constitutional irritation and great discharge of pus. Other cases of a similar nature lately brought under Mr. Birkett's charge, in which the iodine cure has been practised, were the result of common milk abscess, from which the above, as will be seen, differs in some particulars.

Prevention *is* better than cure, and, as we believe, there are few diseases more thoroughly within the reach of prophylactic measures, than is milk abscess, we cannot dismiss the subject without saying a few words on that head. The history of this disease is in most cases easily told. A woman of delicate skin is confined, perhaps for the first time; lactation commences, the cuticular investment of the nipple, irritated by the mouth of the child, becomes cracked and fissured, each application of the infant to the breast occasions torment to the mother, and she avoids it as much as possible. The child is allowed to suck only on the sound side, the milk accumulates in the other breast, and slight inflammation is set up, to be aggravated by the increasing dread on the part of the patient of the natural method of relief, the evacuation of the milk. An abscess is the result. Now, how was all this to be avoided? In most cases pregnant women consult their medical advisers on sundry little points some time before their confinements. Let him on those occasions enquire as to the state of the nipple; and should the skin be found to be delicate, the daily application to the part of an alum wash, decoction of oak bark, or some other astringent, should be recommended. By such means the skin may be hardened, tanned in fact, and rendered just as capable of resisting irritation as that of the finger. In other cases milk abscess depends on the non-development of the nipple. The surgeon should take care that a shield be provided beforehand, and that his patient knows how to use it properly.

But, supposing that, with the greatest care, abscess has proved unavoidable, there are still measures by which the disease may be prevented from passing into the deplorable condition in which we have noted that the patient in the above case came into Mr. Birkett's hands. We have repeatedly heard Mr. Paget observe, that, among his out-patients at St. Bartholomew's, he never has any opportunity for trying the various vaunted injections for the cure of sinuses in the breast, because the latter always heal of themselves. We have very carefully watched Mr. Paget's practice, in which mammary abscesses are very common, and can most fully confirm this statement. His treatment consists in the free evacuation of all collections of matter, and in the internal exhibition of tonics. A generous diet of meat and beer, with full doses of quinine or iron,—such are the remedies under which improvement in the local and general condition of the patient seldom fails to become rapidly manifest. It must, however, be admitted that the applicants, as out-patients, do not include a small class of peracute cases in which the symptoms are often too severe to allow of the patient's leaving her bed. The systematic avoidance of antiphlogistic measures in the treatment of local suppurative inflammations is daily becoming more and more common, and in no respect has modern practice more strikingly advanced than in this. It affords, too, a good illustration of the application of minute pathological research to actual every-day practice. Those of our readers who had the good fortune to hear Mr. Paget's lectures on inflammation, delivered before the College of Surgeons three years ago, will remember how unwillingly the Professor admitted the existence of any increase in formative power in that condition. This view, founded as it was on theoretic reasoning, and microscopic observation of the process, has since been advocated by other pathologists, including some of the German school, and it is interesting to observe how, upon emperical recommendations merely, the line of practice which it would suggest is rapidly coming into vogue.—*London Med. Times and Gaz.*

